# Structure and Dynamics of a Site-Specific Labeled Fc Fragment with Altered Effector Functions

**DOI:** 10.3390/pharmaceutics11100546

**Published:** 2019-10-21

**Authors:** D. Travis Gallagher, Chris McCullough, Robert G. Brinson, Joomi Ahn, John P. Marino, Nazzareno Dimasi

**Affiliations:** 1 Institute for Bioscience and Biotechnology, National Institute of Standards and Technology and the University of Maryland, 9600 Gudelsky Drive, Rockville, MD 20850, USA; chris_r_mccullough@hotmail.com (C.M.); robert.brinson@nist.gov (R.G.B.); john.marino@nist.gov (J.P.M.); 2 Analytical Sciences, AstraZeneca, One MedImmune Way, Gaithersburg, MD 20878, USA; joomee50@gmail.com; 3 Antibody Discovery and Protein Engineering, AstraZeneca, One MedImmune Way, Gaithersburg, MD 20878, USA; nazzareno.dimasi@astrazeneca.com

**Keywords:** antibody drug conjugates, antibody Fc engineering, X-ray crystallography, hydrogen-deuterium exchange mass spectroscopy

## Abstract

Antibody-drug conjugates (ADCs) are a class of biotherapeutic drugs designed as targeted therapies for the treatment of cancer. Among the challenges in generating an effective ADC is the choice of an effective conjugation site on the IgG. One common method to prepare site-specific ADCs is to engineer solvent-accessible cysteine residues into antibodies. Here, we used X-ray diffraction and hydrogen-deuterium exchange mass spectroscopy to analyze the structure and dynamics of such a construct where a cysteine has been inserted after Ser 239 (Fc-239i) in the antibody heavy chain sequence. The crystal structure of this Fc-C239i variant at 0.23 nm resolution shows that the inserted cysteine structurally replaces Ser 239 and that this causes a domino-like backward shift of the local polypeptide, pushing Pro 238 out into the hinge. Proline is unable to substitute conformationally for the wild-type glycine at this position, providing a structural reason for the previously observed abolition of both FcγR binding and antibody-dependent cellular cytotoxicity. Energy estimates for the both the FcγR interface (7 kcal/mol) and for the differential conformation of proline (20 kcal/mol) are consistent with the observed disruption of FcγR binding, providing a quantifiable case where strain at a single residue appears to disrupt a key biological function. Conversely, the structure of Fc-C239i is relatively unchanged at the intersection of the CH2 and CH3 domains; the site known to be involved in binding of the neonatal Fc receptor (FcRn), and an alignment of the Fc-C239i structure with an Fc structure in a ternary Fc:FcRn:HSA (human serum albumin) complex implies that these favorable contacts would be maintained. Hydrogen deuterium exchange mass spectroscopy (HDX-MS) data further suggest a significant increase in conformational mobility for the Fc-C239i protein relative to Fc that is evident even far from the insertion site but still largely confined to the CH2 domain. Together, the findings provide a detailed structural and dynamic basis for previously observed changes in ADC functional binding to FcγR, which may guide further development of ADC designs.

## 1. Introduction

Monoclonal antibodies have become central to anticancer strategies, including their use in delivering cytotoxins to epitopically-targeted malignant cells. Simple in concept, these antibody-drug conjugates (ADCs) are large multi-component constructs requiring an approach that must address several overlapping challenges, including epitope selection, linker design, and control over conjugation. Each of these issues has seen key innovation in the past decade, enabling a recent expansion to over 50 therapeutic candidates currently in clinical trials [1], and five ADC’s approved for clinical use (Mylotar, Adcetris, Kadcyla, Bespona, and Polivy). These ADCs incorporate a wide variety of different cytotoxins derived from bacteria, plants, and animals, and target many types of cancers. Furthermore, they include an assortment of linker designs and conjugation chemistries, as well as a variety of different antibody attachment sites.

Across the vast surface of an IgG molecule, the challenge of selecting effective conjugation sites was addressed initially by attaching toxins to random lysines and transiently-reduced hinge disulfides, which created heterogeneous ADCs. Precise control of the conjugation, and thus of the amount of cytotoxin, was later achieved by enzyme-based approaches and bioorthogonal chemical methods [2]. Site-specific methods also include the simple mutation of a solvent-accessible residue to cysteine (Cys), since IgGs do not normally contain free Cys. Cys can be added either by single-site replacement, by insertion, or by a larger change in sequence. Effective sites of insertion must neither destabilize the IgG nor block antigen-binding or other key functions. Furthermore, insertion must not promote aggregation or instability, and the site must be accessible for conjugation. Three commonly used sites, kappa chain V205 and heavy chain A114 and S239, have been previously studied [3]. For each site, three variants were produced by introducing a Cys before, in place of, or after the selected site, and each of these nine constructs was extensively analyzed. All had solution and thermal stability behavior similar to parent antibodies, especially the simple replacements V205C, A114C, and S239C. In addition, the insertions before and after S239 (i.e., C238i and C239i) showed a new feature: abolition of antibody-dependent cellular cytotoxicity (ADCC) due to non-binding of Fc gamma receptor IIIA (FcγRIIIA). This could be advantageous for ADC therapies, since binding to FcγRIIIA can lead to internalization of the toxin by nontarget cells and consequent side effects.

In order to determine the structural basis for those findings and to provide a framework for further ADC engineering, we have analyzed the structure and dynamics of the Fc-C239i protein by X-ray diffraction and hydrogen deuterium exchange mass spectroscopy (HDX-MS). The crystal structure and dynamics of Fc-C239i with two different adducts, maleimide-PEG8 (polyethylene glycol-8) and cysteine, show how the IgG structure accommodates the insertion and binds the adducts, providing a model for the bound structure of one of the most common ADC linkers. The structures also show how FcγR binding is disrupted via a domino-like perturbation involving conformationally incompatible residues. Implications for molecular energetics and for conjugate design strategies are discussed.

## 2. Materials and Methods

### 2.1. Protein Production

The Fc-C239i protein used for X-ray crystallography was made recombinantly in mammalian cells. The construct had the leader peptide sequence (MDMRVPAQLLGLLLLWLPGARC) for extracellular secretion [4]. The primary amino acid sequence of the Fc-239i is:

DKTHTCPPCPAPELLGGPS**C**VFLFPPKPKDTLMISRTPEVTCVVVDVSHEDPEVKFNWY

VDGVEVHNAKTKPREEQYNSTYRVVSVLTVLHQDWLNGKEYKCKVSNKALPAPIEKTI

SKAKGQPREPQVYTLPPSREEMTKNQVSLTCLVKGFYPSDIAVEWESNGQPENNYKTT

PPVLDSDGSFFLYSKLTVDKSRWQQGNVFSCSVMHEALHNHYTQKSLSLSPGK

The inserted cysteine after position 239 is underlined and bolded.

The Fc-C239i DNA expressing cassette was cloned into a proprietary mammalian expression vector [4]. The expression vector was transfected in CHO-G22 cells and expression was carried out in a proprietary CHO-V2 medium at 37 °C, 5% CO_2_, supplemented with 25 μM l-methionine sulfoximine, and 100 μg/mL hygromycin [4]. The culture medium was collected 14 days after transfection, and purification was carried out using standard protein A affinity chromatography. After purification, the Fc-C239i was dialyzed in PBS (phosphate buffered saline) pH 7.2, 1 mM EDTA (ethylenediamine tetraaceticacid). The purified Fc-C239i had a monomeric content of 99% as determined by analytical size-exclusion chromatography (data not shown).

### 2.2. Sample Preparation for Crystallography

Recombinant expression of Fc-C239i resulted in a homogeneous adduct with cysteine present in the culture medium as demonstrated using non-reducing liquid chromatography mass spectrometry (data not shown). Cysteine adduction to free cysteines engineered into antibodies has been described previously [3,5]. The Fc-C239i cysteine adduct was used for crystallization without any further purification. The Fc-C239i-maleimide-PEG8 adduct was prepared essentially as reported previously [3,5]. The Fc-C239i cysteine adduct was reduced using 40 mol equivalents of TCEP (tris(2-carboxyethyl)phosphine) in PBS pH 7.2, 1 mM EDTA for 3 h at 37 °C. Following 2× dialysis in PBS pH 7.2, 1 mM EDTA at 4 °C using 10,000 kDa molecular weight cutoff dialysis cassettes, 20 mol equiv of dehydroascorbic acid was added for 4 h at 25 °C. The solution was filtered through a 0.2 μm syringe filter, and 10% (*v/v*) DMSO, and 4 equiv of maleimide-PEG8 (Quanta BioDesign, Plain City, OH, USA) were added, followed by incubation at room temperature for 30 min under gentle rotation. The conjugation was quenched by the addition of 4 mol equivalents (over maleimide-PEG8) of *N*-acetyl cysteine. The conjugation process resulted in ~5% of aggregate formation. Macromolecular aggregates and conjugation reagents, including cysteine quenched maleimide-PEG, were removed using ceramic hydroxyapatite type II chromatography (CHT) as described previously [3,5]. The conjugate was dialyzed into 25 mM histidine-HCl pH 6.0 for storage and analysis. Homogeneous conjugation was demonstrated using non-reducing and reducing liquid chromatography mass spectrometry (data not shown). Figure S1 shows diagrams of both adducts.

### 2.3. Crystallography and Structure Analysis

The constructs were prepared for crystallization by buffer exchange into 25 mM KCl, 10 mM sodium acetate, pH 5.0, and concentration to 15 mg/mL. Crystal screening against about 300 conditions yielded clusters of rectangular bars from several low-pH conditions that were optimized to 18% polyethylene glycol 4000, 150 mM potassium acetate, pH 8.0. Single crystals were harvested and cryoprotected by immersion for 3 s in the above mixture with glycerol added to 18%. Crystals were then frozen by plunging into liquid nitrogen and kept at a cryogenic temperature through data collection at beamline 19-ID of the Advanced Photon Source at Argonne National Labs. The cysteine adduct diffracted to 0.23 nm and the maleimide adduct diffracted to 0.27 nm (see diffraction statistics in Table S1).

Both structures were built starting from a pared-down wild-type human Fc structure derived from entry 5VGP in the PDB (Protein Data Bank) [6]. The starting model was prepared by removing its N-terminal strand, nearby loops, glycans, and water molecules. These parts were then rebuilt to difference electron density maps in an iterative refinement process, utilizing the crystallographic refinement suite CCP4 [7] and the program COOT [8]. Each final structure contained a complete Fc fragment, with the residues 237 through 445 of two heavy chains, denoted A and B. Each chain has an asparagine-linked octasaccharide glycan bound to asparagine 297, and several dozen bound water molecules, and a specific adduct (cysteine or maleimide-PEG8) bound to the inserted cysteine 239i. Refinement of the structure with the cysteine adduct led to the final deposited model PDB:6P6D. All figures were made using Pymol [9]. The structure with the maleimide adduct, due to its lower resolution and marginal order for the maleimide-PEG8, was not deposited.

### 2.4. Hydrogen Deuterium Exchange Mass Spectrometry

The peptide coverage maps for both samples (with no adduct and with the maleimide-PEG8 adduct) were obtained from the undeuterated controls as follows: 2.5 μL of sample was diluted with 47.5 μL of buffer solution at room temperature and followed with the addition of 100 μL of ice-cold quench (4 M guanidine-HCl, 250 mM TCEP at pH 2.5). Quenched samples were immediately injected into Waters nanoACQUITY UPLC system [10]. The online pepsin digestion was performed using a 2.0 × 30 mm Ezymate BEH pepsin column (Waters) for 4 min in 0.15% formic acid in H_2_O at a flow rate of 100 µL/min at 25 °C and the peptic peptides were trapped and desalted online using an ACQUITY UPLC BEH C18 1.7 µm VanGuard Pre-column (Waters, Milford, MA, USA) at 0 °C. The peptides were eluted into a 1.0 × 100 mm ACQUITY UPLC BEH C18 column (Waters) held at 0 °C and were separated at a flow rate of 40 µL/min in a step-gradient; 8–15% acetonitrile gradient in the first min followed by 7 min linear acetonitrile gradient (15–32%) and held at 40% acetonitrile for 1 min. The eluent was directed into a Waters Xevo Q-Tof G2 XS mass spectrometer with electrospray ionization and lock mass correction. Peptic peptides were acquired in MS^E^ mode and identified using Waters ProteinLynx Global Se(PLGS) 3.0 [11,12,13] and the PLGS outputs were processed in Waters DynamX 3.0 software (Waters, Milford, MA, USA).

For deuterated samples, the dilution and quench steps were the same as for undeuterated controls. The labeling solution was introduced to the protein stock solution in 20-fold excess, incubated at room temperature for 10 min followed by a quenching step as described above. The deuterium uptake in Da and percentage were determined from the mass shift between deuterated and undeuterated peptide ions pertaining to the intensity-weighted center mass using DynamX software. For each peptide, the relative deuterium uptake was plotted without back-exchange correction. The average back-exchange was observed to be 24% using a highly deuterated 9-peptide standard. This level of back-exchange is similar to previous reports [14,15,16]. For both the HDX and diffraction-based dynamics comparisons, wild-type measurements for each amino acid were subtracted from the corresponding measurements for Fc-C239i.

## 3. Results

The structural implications of modifying a human IgG1 Fc domain for the purposes of developing an ADC through cysteine insertion were explored. In the two structures reported herein, a cysteine was inserted after Ser 239 (Fc-C239i) (for full sequence, see Section 2). The location of this insertion was just after the hinge within the first few residues of the Fc. The local sequence is LLGGPS**C**VFLF with the GG motif occurring at the end of the hinge in the wild-type Fc domain. The two reported structures feature two different covalent adducts bound to the inserted cysteine. In the first structure, which diffracted to 0.23 nm resolution and was deposited to the Protein Data Bank as 6P6D, the adduct was an additional cysteine bound to Cys 239 by a disulfide. In the second structure, the adduct was a maleimide group with an octa-ethylene glycol (PEG8) tail.

### 3.1. Structure of Fc-C239i

Crystals grew as rectangular prisms with favorable dimensions for synchrotron data collection, about 60 µm × 150 µm × 400 µm. The cysteine-adduct and maleimide-adduct crystals diffracted to 0.23 nm and 0.27 nm resolutions, respectively. Both samples crystallized in a form that is isomorphous to over 30 previous structures of human Fc fragments (with various isotypes, mutations, and glycoforms) in the Protein Data Bank [6], including the wild-type reference structure 5VGP [17]. In the packing arrangement of all crystals of this form, the hinge is disordered and omitted from the refined model. The two chains, though chemically identical, have slightly different packing contacts and slightly different conformations in some surface loops. However, for the region of the cysteine insertion, and all the regions described in this report, there are no significant differences between the two chains.

**Figure 1:**
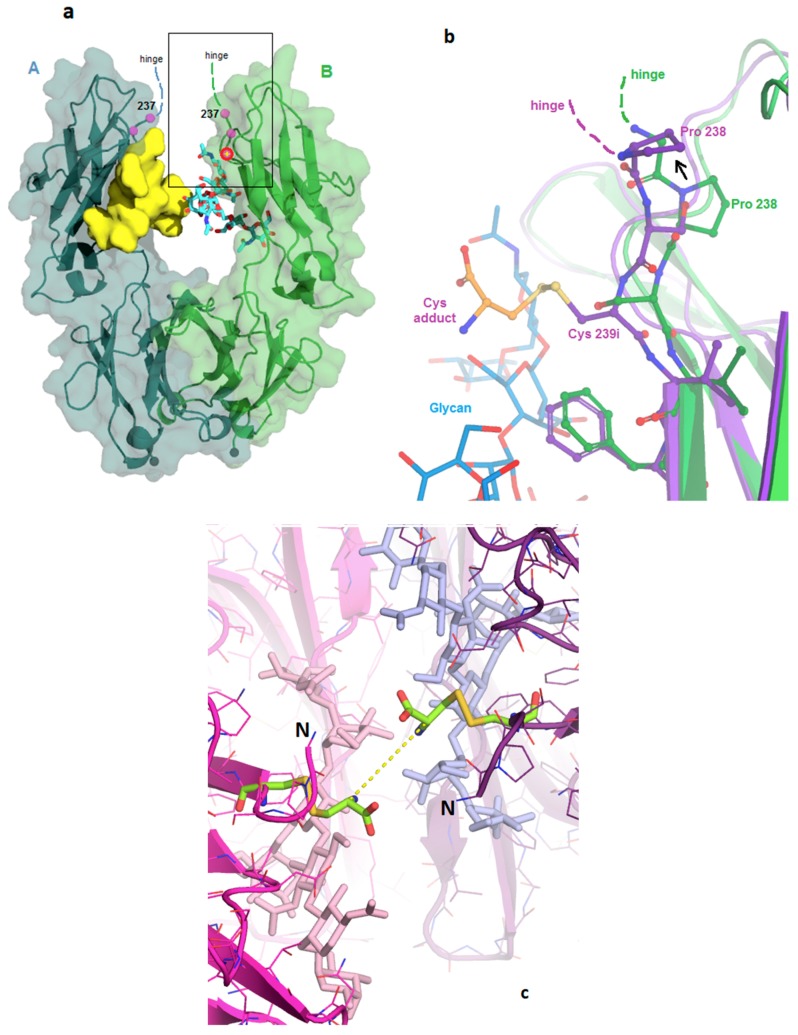
Fc-C239i structure. (**a**) Wild-type Fc showing the area of the inserted cysteine. Glycans are shown using a yellow surface for the A-chain and cyan sticks for the B-chain. The location of glycine 237 is indicated in both chains, and the location of serine 239 in the B-chain is shown with a yellow cross and red circle. The rectangle corresponds to the zoomed-in region of (**b**). (**b**) Structural effects of the inserted cysteine. Superposition of the C239i protein (purple, PDB:6P6D) onto wild-type (green, PDB:5VGP) in the region of the inserted cysteine, to which an extra cysteine (adduct, gold colors) is bound by a disulfide. The inserted cysteine 239i occupies the location held by Ser 239 in the wild-type. This displaces the serine upward, causing Pro 238 also to shift upward (arrow). (**c**) View down the molecular dyad of Fc-C239i into the solvent cleft, with the A-chain in magenta and the B-chain in purple. All four Cys residues are colored green (each chain has its Cys 239i insertion, and each of these carries a disulfide-linked cysteine adduct). N termini (labeled with ‘N’) are to the left and right of the center. Glycans are visible just beyond the cysteines (pink and blue sticks). The central dashed line shows the 0.7 nm distance between the alpha carbons of the two adducts across the dyad.

In the reference Fc structure 5VGP, the region of the Fc corresponding to the cysteine insertion point comprises the first strand of its antiparallel beta fold and has extensive hydrogen bonding to the adjacent strand in the CH_2_ domain. The 6P6D crystal structure shows that the modified Fc maintains the H-bonding, and the structural effects of the insertion are limited to the first three residues of the Fc. As shown in Figure 1a,b, the inserted cysteine replaces Ser 239 and causes a 1-residue upward shift of Ser 239, Pro 238, and Gly 237. This enables Val 240 and all subsequent residues to maintain their wild-type locations. The new cysteine side chain points outward from the protein, into the solvent cleft between the A and B subunits. The change effectively replaces the side chains at two well-ordered sites (238 and 239), without disrupting any H-bonds. The new cysteine thiol appears accessible for conjugation to a linker/payload (Figure 1c). The side chain of Ser 239 is now observed in the pocket formerly occupied by Pro 238, which has adequate volume but no H-bonding partner for the serine hydroxide. This slightly unfavorable location for the serine appears to cause the main chain to bend outward slightly (Figure 1b). A wave of displacement then puts Pro 238 into the place normally occupied by Gly 237, formerly the last residue of the wild-type hinge. Since there are no steric constraints against the proline here, the upward-shifting of the insertion results in adding one residue to the length of the hinge.

### 3.2. Structures of the Fc-C239i Adducts

The observed location of Cys239i and the upward shift of residues Pro 238 and Ser 239 are similar in both chains of both adduct structures. In structure 6P6D, the two cysteine adducts appear to have the same conformation, with their alpha carbons 0.7 nm apart across the cleft (dashed line in Figure 1c). These blocking cysteines provide an approximate model for the steric volume available to the first link of more elaborate conjugates and payloads. Maleimides are known to undergo gradual hydrolysis to a linear form, which stabilizes the conjugation, and the electron density is better fit by this linear form than by their five-membered ring form. Beyond that, observed density for the maleimide groups is weak and consistent with disorder. Although the crystallized sample included PEG8 linkers beyond the maleimides, these were not observed.

### 3.3. Fc-C239i Dynamics

**Figure 2:**
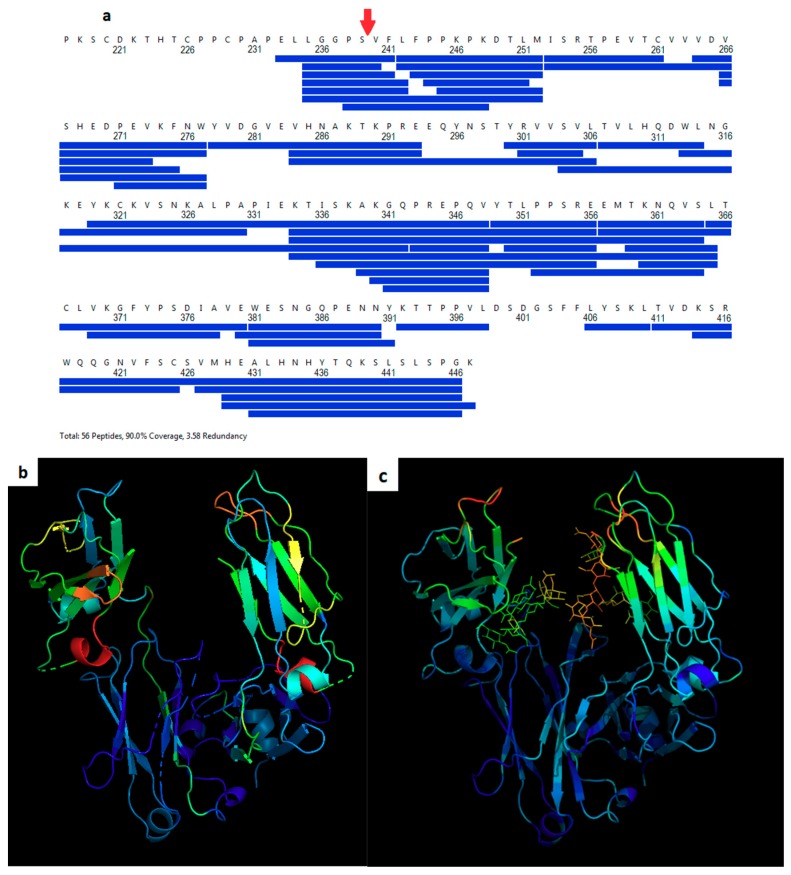
Dynamics of Fc-C239i. (**a**) Coverage map for hydrogen-deuterium exchange. The insertion site after Ser 239 is indicated with a red arrow. (**b**) Heat map based on hydrogen-deuterium exchange (HDX) mass spectrometry. Colors show the range of measured mobility increases, using a spectrum from blue for the lowest values to red for the highest. Observed percent changes in HDX range from −2% to +19% (see Figure S2). Warmer colors represent regions of higher differential exchange (mutant minus wild-type HDX measurements of the peptide fragments provided full coverage of the backbone sequence with the exception of residues 253, 278, 392, and 399–406 (shown with dashes)). (**c**) Heat map based on crystallographic thermal displacement values (B-factors). Warmer colors show areas where the insertion increases B values relative to wild-type. The crystallographic data also include the glycans.

Hydrogen-deuterium exchange (HDX) was measured for the Cys-239i inserted protein with conjugated maleimide-PEG8 and compared to the wild-type Fc control. Peptide fragmentation gave 95% coverage, beginning with Ser 239 (Figure 2a). While the CH3 domain showed only background differential HDX values of a few percent (Figure 2b, Figure S2), all CH2 peptides showed increased HDX, indicating that the insertion generally increases the solvent accessibility of the CH2 domain’s amide hydrogens. The greatest increases in HDX (red and orange regions in Figure 2b) occurred in exposed loops that are far from the domain’s core. Crystallographic temperature factors provide an orthogonal measure of protein dynamics, as they report the magnitude of atomic disorder or motion within the crystal (Figure 2c). As with HDX, the crystal analysis subtracts wild-type (5VGP) mobility values from those of Fc-C239i (with adduct, i.e., 6P6D) to indicate the mobility effect of the insertion. The crystallographic measurements include the glycans and give distinct data for the two chains, subject to the artifact of restraint at crystal contacts. As with HDX, the crystal-based thermal factors show no significant changes in the CH3 domain, while most of the CH2 domain appears destabilized, especially loops near the insertion (Figure 2c).

## 4. Discussion

**Figure 3:**
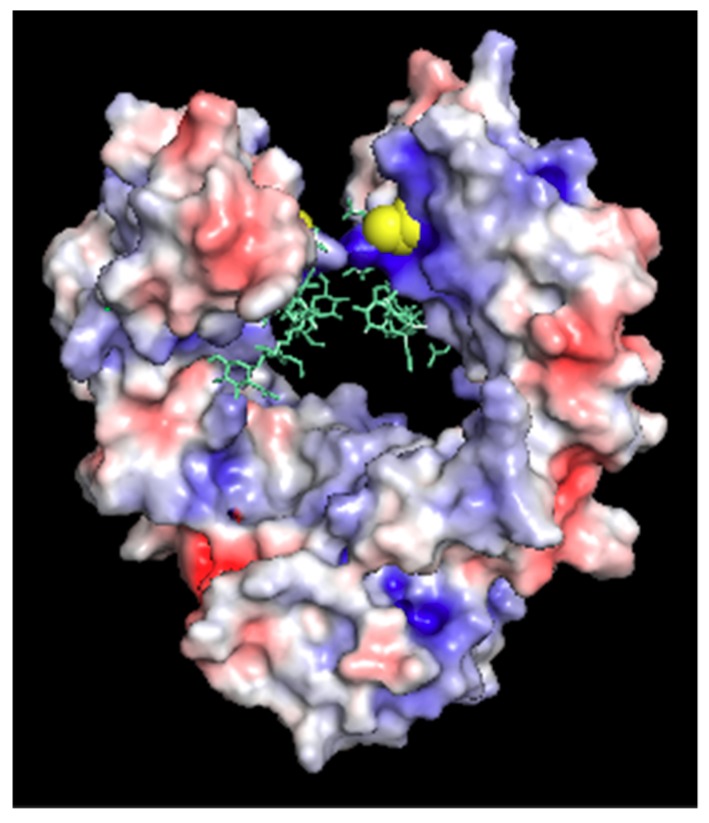
Electrostatic surface potential of Fc-C239i, calculated using the adaptive Poisson-Boltzmann program within Pymol. Glycans are added using cyan sticks, and the inserted Cys is emphasized with yellow spheres. Note the blue area (electropositive) around the C239i residue.

The reported structure of the human Fc cysteine insertion mutant C239i enables correlation between atomic-level features and biological effects. The described structures and dynamics offer implications for ADC functional design, in particular for the location of the conjugation site on the antibody. The overall stability of the C239i construct is consistent with its minimal disruption of the tertiary structure downstream of the insertion. The observed main-chain perturbation is a local effect that alters the locations of Ser 239 and Pro 238, effectively making the hinge one residue longer. The location of the new cysteine side chain is within the Fc cleft. It is close to the glycan (about 0.5 nm), and the distance between the two C239i residues across the cleft (A-chain to B-chain) is 1.1 nm. This location appears to be chemically accessible, in that it conjugates with a linker/payload as demonstrated by preparation of several ADCs, one of which is in clinical investigation [3,5,18,19] while being somewhat protected from access by other proteins. Due primarily to the nearby side chains of arginine 301 and lysine 334, the local environment is electropositive (Figure 3). Overall, these observed effects appear to be the protein’s lowest-energy way to adapt to the inserted cysteine, enabling it to maintain its native stable fold.

This structural alteration is apparently energetically preferred over other possible ways to accommodate the insertion into the Fc fold. For example, one could imagine all wild-type residues remaining in their places, and the new cysteine existing as an outward bulge between Ser 239 and Val 240. However, this would be precluded by the large strain it would place on the two consecutive peptide bonds. Alternatively, it is possible that the new cysteine could replace Val 240, and cause a downward, instead of upward, shift in the local strand. However, this would perturb more residues, including several hydrophobic side chains (e.g., Val 240 and Leu 242) that fit snugly into the core of the folded domain, which would be highly unfavorably energetically. The specific manner in which the structure incorporates the extra residue, with a ripple effect to the end of the beta strand, offers a simple predictive model for other possible insertions within beta sheet strands [3]. Most of the IgG’s beta strands are six to eight residues long, so a single residue insertion can induce a shift of up to a few residues, to resolve the ‘bump’ into an adjacent loop. The side chains involved will impact the energetics; since beta structure typically involves alternating hydrophobic and hydrophilic side chains, inserting two residues may be less disruptive than inserting one.

Increases in protein mobility were measured by two distinct methods, each indirect and subject to their own experimental artifacts. Dynamics measurement by HDX rely on inferring protein mobility from hydrogen-deuterium exchange, whereas the local protein structure can modulate this relationship, due to protection, water structure, and conformational factors. Dynamics measurement by crystallographic disorder parameters (B-factors) is also subject to nonlinear local structure effects, as well as to physical restraint at crystal contacts. In the crystal structure, about 10% of the protein atoms are solvent-occluded and dynamically restrained by the crystal contacts [20]. The greatest increase in B-factors are for the loop that includes Leu 328 (upper left in Figure 2c), apparently destabilized by loss of contact with Pro238, which was moved away by the insertion (see also Figure 1b). Despite the different methods, the resulting dynamics profiles appear largely similar (Figure 2b,c). The Pearson correlation between the two different mobility-per-residue profiles is 0.31 (for the X-ray profile, the two chains were averaged). Additionally, for each method, the correlation was calculated between the residue mobility increase and the distance from the insertion site. These two correlations are –0.48 and –0.74, for HDX and X-ray, respectively, consistent with the fact that the dynamics perturbations from the insertion are mostly local within the CH_2_ domain. This metric is expected to be negative, based on a simple model in which perturbations propagate outward from the insertion site and diminish at large distances.

The observed structure and dynamics are consistent with previously reported calorimetric findings for this protein, and also help to explain DSC (differential scanning calorimetry) results for other constructs. As previously shown [3], the simple S239C replacement did not affect the DSC profile, consistent with its replacing only one solvent-exposed atom. In contrast, the C239i insertion additionally causes two side chains (Ser and Pro) to move into new environments. The energy cost of the insertion can be roughly inventoried as the cost of putting the Ser 239 side chain into the proline pocket, plus the cost of putting the proline out of its pocket, into the solvent, and pushed into the hinge. The appearance of a small new DSC melting transition at 63 °C (along with the main transition at 73 °C characteristic of the native antibody) likely reflects this destabilization. While C239i produces the local sequence GPSCV, the C238i insertion produces the sequence GPCSV. Thus, the 238i insertion can be predicted to leave Ser 239 in place, to locate the new Cys in the proline’s place, and again to put the proline out into solvent as a new hinge residue. This predicted perturbation is almost identical to that observed in 6P6D, consistent with an almost identical DSC profile [3].

**Figure 4:**
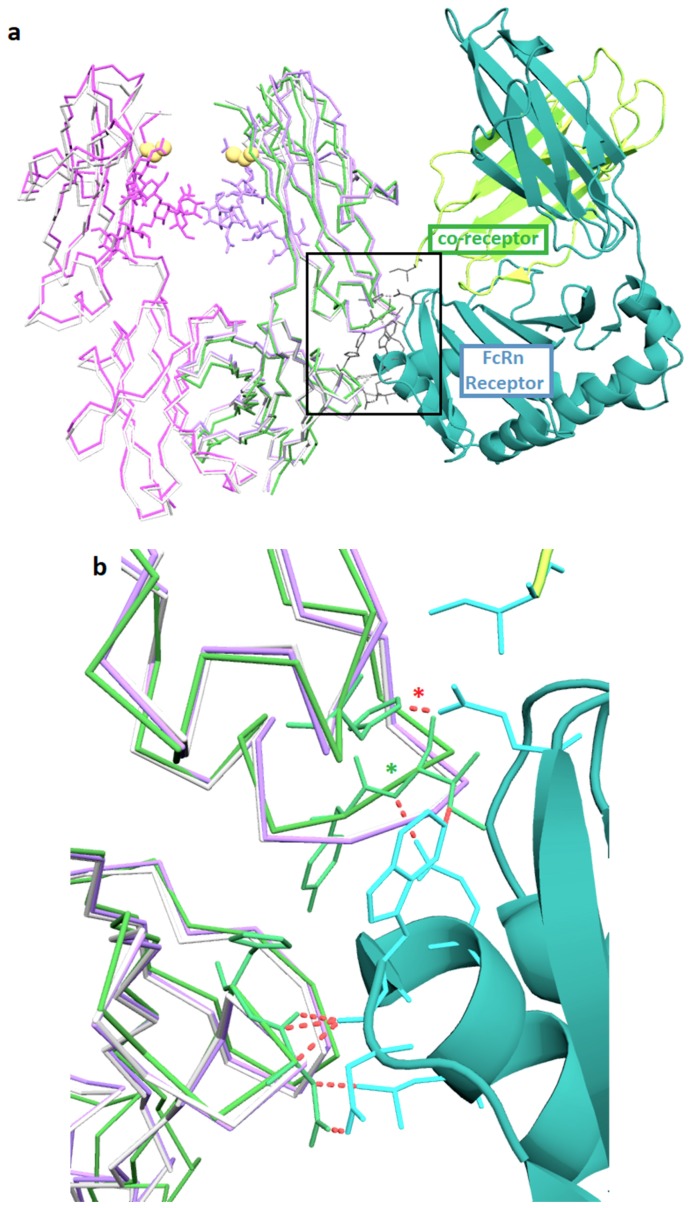
FcRn binding interface. (**a**) Superposition of Fc-C239i (A and B-chain in magenta and purple, with sticks for glycans) onto the complex (PDB: 4n0u) of Fc-wt (wild-type) (green) with the FcRn receptor (cyan and yellow cartoon). Yellow spheres show the location of the inserted cysteine in each chain. Also superposed is the uncomplexed wild-type (5VGP) in light gray. Gray sticks show key sidechains in the receptor interface, which is over 3.0 nm from the insertion. (**b**) Close up of the interface (box in (**a**)). The pH dependence of binding is due to the histidine-containing ionic interaction indicated by the red asterisk. The green asterisk shows where a central interface loop is apparently bent upward by about 0.1 nm in the complex. The interface region appears structurally unaffected by the insertion (purple overlays closely with gray), consistent with biophysical measurements showing that FcRn binding is maintained.

The neonatal Fc receptor (FcRn) extends the half-life of IgG by reducing lysosomal degradation in endothelial cells [21]. The location of the FcRn binding on the IgG is at the Fc elbow, which is about 3 nm away from the C239i insertion site. The structure of Fc-C239i, compared with the previously described complex of Fc with FcRn [22], indicates that there are no significant perturbations in this region (Figure 4), consistent with previous measurements showing that binding is unaffected [3]. This is important for ADC pharmacokinetics, as FcRn has a protective role, stabilizing antibodies and ADCs against clearance from the blood [21]. Another potentially important factor in serum stability of the ADCs is the electrostatic potential of the modified antibody. Figure 3 shows the electrostatic surface of Fc-C239i, which has an electropositive patch near the insertion site, largely due to the proximity of lysine 334. It has previously been reported that an electropositive neighborhood around the conjugation site promotes stabilization of the thiosuccinimide adduct when maleimide-based linkers are used [23].

**Figure 5:**
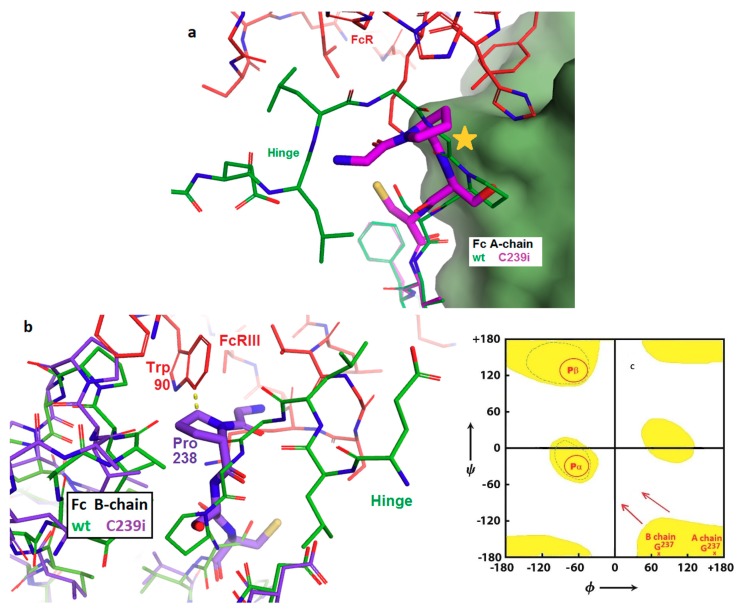
Structural basis for prevention of antibody-dependent cellular cytotoxicity (ADCC) through loss of FcγRIIIA binding. (**a**) Superposition of Fc-C239i (purple) onto the Fc-wt (green) in its complex (PDB: 3ay4) with the FcγRIIIA receptor (red) in the area of the insertion in the A-chain. The inserted cysteine is just below the center. The star indicates the location of the highly extended Gly 237 in Fc-wt, which is occupied by Pro 238 in the mutant. The proline is unable to adopt the glycine’s extended conformation, changing the path of the polypeptide and disrupting the interface. (**b**) As with the A-chain in Figure 4a, the B-chain interface is likewise disrupted by the inability of Pro 238 to conformationally replace Gly 237. In addition, the location of Trp 90 in the 3ay4 complex is within 0.3 nm of the popped-up proline (dashed line), causing further destabilization of the interface. Colors are given as in (**a**). (**c**) Ramachandran diagram showing energetically-allowed regions for glycine (yellow) and for proline (red circles). The Fc-wt A-chain and B-chain locations of Gly 237 are shown at the lower right; proximity to the corners correlates with their extended conformation as observed in the 3ay4 complex. The arrows indicate that when proline displaces glycine, the conformation is forced to change. Due to steric constraints from adjacent parts of the Fc, both prolines are forced into the sharply bent P-alpha conformation, disrupting receptor binding.

In contrast, the binding interface between Fc and the Fc-gamma receptors (types I, II, and III, which all use a conserved domain to bind Fc) is structurally close to residue 239 (PDB: 3ay4, [24]) and is disrupted by the C239i insertion (Figure 5). This interface involves mainly one domain of the FcγR structure, but it involves both chains of the Fc. Moreover, it includes extensive contacts involving the Fc glycan and the FcR glycan bound to its Asn 162. This glycan–glycan contact appears to regulate the ‘fucose effect’, whereby removal of fucose from IgG causes a dramatic increase in ADCC [25]. The observed rearrangement of local structure adjacent to the C239i insertion provides an explanation for the previously reported loss of FcγR binding by Fc-C239i, by placing Pro 238 into the location where Gly 237 normally has an extended conformation that is incompatible with proline (Figure 5). Glycine is well-conserved in this position, consistent with an important role in receptor binding and signaling. Although the receptor interface is asymmetric and features distinct contacts to the two Fc chains, both instances of Gly 237 adopt the extended conformation in the wild-type interface, so that the C239i insertion disrupts the binding to both Fc chains.

Fc gamma receptors of several types, including both activating and inhibitory, are present on cells of hematopoietic origin and play various signaling and regulatory roles, including the stimulation of antibody-dependent cellular cytotoxicity (ADCC). These receptors bind to IgG with an elaborate interface that includes the bottom of the hinge and the upper cleft of the Fc. Crystal structures of these complexes show binding to both chains of the Fc, including direct interactions between the Fc glycan and a glycan carried by the receptor. It is this glycan–glycan contact that explains the dramatic stimulation of ADCC produced by antibodies that lack fucose. Structure 6P6D shows the basis for the previously reported complete loss of FcγR binding of, and ADCC function by, Fc-C239i [3]. The upward shift of the Gly-Pro-Ser motif at the N-terminus of the Fc disrupts the interface by placing Pro 238 in the location previously held by Gly 237 in a highly extended conformation (Figure 5). The extended, nearly straight, conformation is accessible only to glycine and least of all to proline. The energy gain in forming the FcγR complex is estimated to be about 7 kcal/mole [20], while the energy cost of forcing proline into the extended conformation is estimated at 20 kcal/mole [26]. This appears to be a case in which disruption of a key biological process can be attributed to conformational strain at a specific residue.

**Figure 6:**
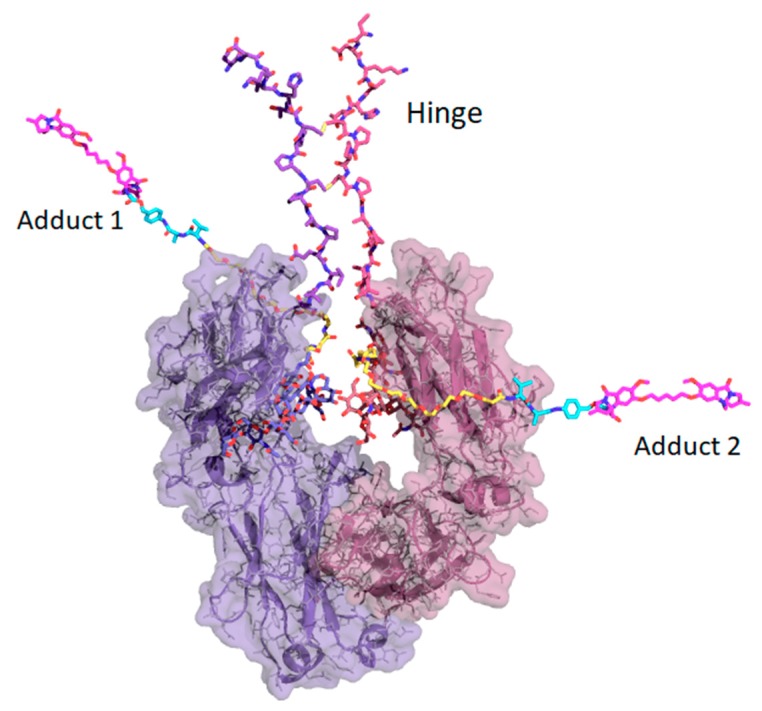
Structure of Fc-C239i with the hinge and full-length conjugates modeled using tesirine (SG3249) added as hypothetical models. Glycans are shown in their observed locations (blue and grey, in the lower cleft). The hinge (upper center) is drawn with its two disulfides, which constrain it and limit the space between the two polypeptides. The two conjugates are attached to the C239i residues in the cleft. Each conjugate is colored with its first portion (maleimide and PEG8 linker) in yellow, and its middle portion (cathepsin cleavage site and linkage-dependent para amino benzoic acid spacer) in cyan. Each conjugate ends with a cytotoxic DNA-modifying pyrrolobenzodiazepine dimer (magenta color). Conjugates are modeled in an extended conformation for clarity. Note that the extended conjugate is long enough (about 6 nm) to wrap around the CH2 domain, and to present its central protease site to cathepsin for cleavage. Although the two conjugates are shown projecting outward on opposite sides of the Fc cleft, it appears possible that they could emerge on the same side, where they could interact with each other.

To explore the implications of the C239i site for drug conjugation, Figure 6 shows a hypothetical model of the protein carrying full-length conjugates at both insertion sites using tesirine (SG3249) as the model payload [3]. The model also includes the hinge, since it is close enough to affect conjugation geometry. The gap or cleft between the two CH2 domains, occupied by the glycans, is narrow, varying by only a few tenths of a nanometer in width across dozens of Fc crystal structures, and is constrained by the dimer geometry as well as by the hinge disulfides. Figure 6 shows that each of the conjugates could in principle project out from the cleft on either side, ending up on the same side or on opposite sides. It would however be difficult for a bound conjugate to pass through the cleft from one side to the other. Thus, it appears that for a C239i antibody carrying two conjugates, the two conjugates could project on opposite sides, or on the same side of the Fc, where they could interact.

## 5. Conclusions

Insertion of cysteines, rather than site-specific mutations, into a defined region of an antibody Fc domain affords the robust preparation of site-specific antibody drug conjugates (ADCs) as demonstrated by the preparation of several ADCs, one of which is under clinical investigation [3,5,18,19]. The structures described herein show that key structural features for binding to FcRn, which protect antibodies and ADCs from catabolism, are maintained between the cysteine inserted Fc and wild-type Fc. Moreover, the C239i structure reveals the mechanism for the reduced binding of the C239i to Fcγ receptors (FcγRs). This could be a desired component of ADCs since emerging data suggest that potential dose-limiting toxicity of ADCs comes from the non-specific uptake of the ADCs through the binding of the ADCs Fc to cells expressing FcγRs [27].

## Supplementary Materials

The following are available online at https://www.mdpi.com/1999-4923/11/10/546/s1. Figure S1: Diagrams of the adducts, Figure S2: HDX heat map by sequence, Table S1: X-ray diffraction statistics.
