# Supplementary material for: Structure and Dynamics of a Site-Specific Labeled Fc Fragment with Altered Effector Functions
Source: Pharmaceutics. 2019 Oct 21;11(10):546. doi: 10.3390/pharmaceutics11100546 (PMC6835914; doi:10.3390/pharmaceutics11100546)
Supplement: Supplementary file 1 [file pharmaceutics-11-00546-s001.pdf]

pharmaceutics

Supplementary Materials: Structure and Dynamics of a Site-Specific Labeled Fc Fragment with Altered Effector Functions

D. Travis Gallagher, Chris McCullough, Robert G. Brinson, Joomi Ahn, John P. Marino and Nazzareno Dimasi

X-ray Diffraction Statistics

Data collection statistics for the highest resolution shell are given in parentheses.

Table S1. Diffraction and Refinement Statistics for ADC-Maia constructs.

Diffraction	Maia-Cys	Maia-Maleimide
Space group	$P2_12_12_1$	$P2_12_12_1$
a, *b*, *c* (nm)	50.19, 81.06, 136.92	49.87, 80.30, 133.64
Resolution range (nm)	3.000–0.230 (0.238–0.230)	3.000–0.272 (0.285–0.272)
R_{merge} [1]	0.070 (0.241)	0.091 (0.300)
Resolution (nm) at which $\langle I/\sigma(I)\rangle = 3$	0.232	0.301
Completeness (%)	95.0 (85.9)	99.1 (99.7)
Redundancy	3.6 (3.2)	3.6 (3.8)
Refinement		
Residues	418	418
Sugar units	16	16
Nonhydrogen protein atoms	3630	3634
Water molecules	372	301
R_{work}/R_{free}	0.224/0.274	0.244/0.299
Overall Mean *B*-value (nm^2)	0.686	0.777
Bond lengths rmsd from ideal (pm)	0.9	0.8
Bond angles rmsd from ideal (degrees)	1.36	1.47

[1] $R_{merge} = \Sigma|I - \langle I\rangle|/\Sigma I$ where $\langle I\rangle$ is the mean of symmetry-related reflection intensities.

Supplementary Figures

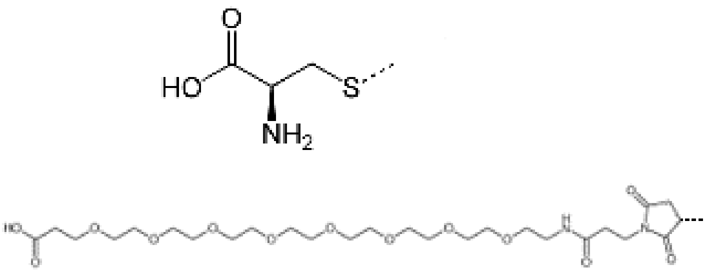

Figure S1. Diagrams of the cysteine adduct (top) and the maleimide-PEG8-acid adduct (bottom). For both adducts, the dashed bond on the right links the adduct to the sulfur of C239i.

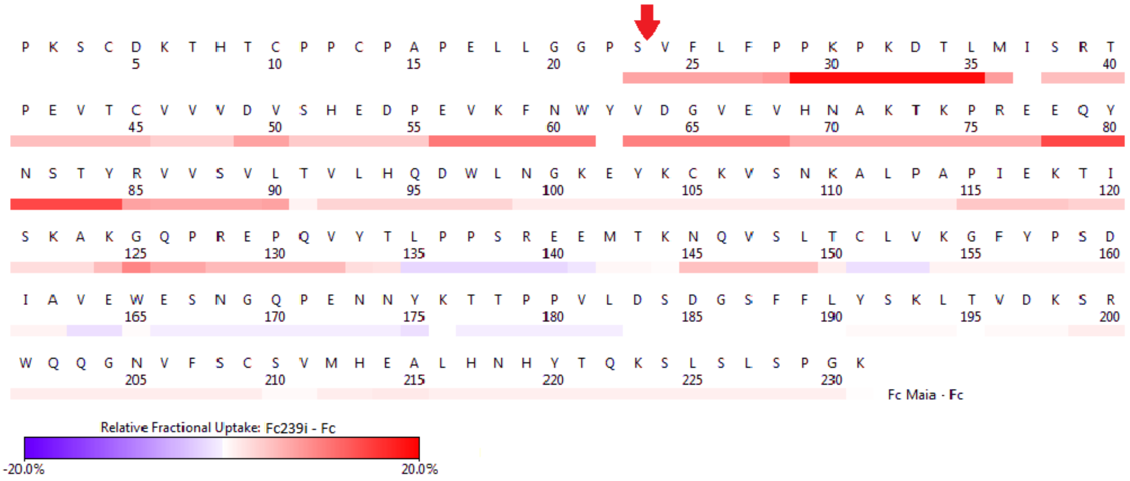

Figure S2: HDX heat map by sequence. Red indicates increased HDX in Fc-C239i relative to Fc. Red arrow indicates the insertion site as in Figure 2a.
